# Efficacy and safety of canakinumab in adolescents and adults with colchicine-resistant familial Mediterranean fever

**DOI:** 10.1186/s13075-015-0765-4

**Published:** 2015-09-04

**Authors:** Ahmet Gül, Huri Ozdogan, Burak Erer, Serdal Ugurlu, Ozgur Kasapcopur, Nicole Davis, Serhan Sevgi

**Affiliations:** Istanbul Faculty of Medicine, Department of Internal Medicine, Division of Rheumatology, 34093 Fatih Istanbul, Turkey; Cerrahpasa Faculty of Medicine, Department of Internal Medicine, Division of Rheumatology, Istanbul, Turkey; Department of Pediatrics, Istanbul University, Istanbul, Turkey; Novartis Pharmaceutical Corporation, East Hanover, NJ USA; Novartis Pharmaceutical Corporation, Istanbul, Turkey

## Abstract

**Introduction:**

This open-label pilot study aimed to investigate the efficacy of canakinumab in colchicine-resistant familial Mediterranean fever (FMF) patients.

**Method:**

Patients with one or more attacks in a month in the preceding 3 months despite colchicine were eligible to enter a 30-day run-in period. Patients who had an attack during the first run-in period advanced to a second 30-day period. At the first attack, patients started to receive three canakinumab 150 mg subcutaneous injections at 4-week intervals, and were then followed for an additional 2 months. Primary efficacy outcome measure was the proportion of patients with 50 % or more reduction in attack frequency. Secondary outcome measures included time to next attack following last canakinumab dose and changes in quality of life assessed by SF-36.

**Results:**

Thirteen patients were enrolled in the run-in period and 9 advanced to the treatment period. All 9 patients achieved a 50 % or more reduction in attack frequency, and only one patient had an attack during the treatment period. C-reactive protein and serum amyloid A protein levels remained low throughout the treatment period. Significant improvement was observed in both physical and mental component scores of the Short Form-36 at Day 8. Five patients had an attack during the 2-month follow-up, occurring median 71 (range, 31 to 78) days after the last dose. Adverse events were similar to those observed in the previous canakinumab trials.

**Conclusion:**

Canakinumab was effective at controlling the attack recurrence in patients with FMF resistant to colchicine. Further investigations are warranted to explore canakinumab’s potential in the treatment of patients with colchicine resistant FMF.

**Trial registration:**

ClinicalTrials.gov NCT01088880. Registered 16 March 2010.

**Electronic supplementary material:**

The online version of this article (doi:10.1186/s13075-015-0765-4) contains supplementary material, which is available to authorized users.

## Introduction

Familial Mediterranean fever (FMF), the most common form of hereditary autoinflammatory disorder, is characterized by recurrent attacks of fever with serosal or synovial inflammation, generally lasting 12 to 72 hours [1]. It has also been associated with increased risk of secondary amyloidosis, mainly affecting renal and vascular function in untreated or insufficiently treated patients with FMF.

Colchicine, the standard of care for patients with FMF, has been considered as safe and effective in the majority of the patients for reducing both the frequency of inflammatory episodes and the risk of developing amyloidosis [2–4]. However, there are currently no effective and approved alternatives for FMF patients who are intolerant to colchicine, and dose reductions due to adverse effects may result in diminished efficacy. In addition, approximately 5−10 % of patients with FMF continue to have frequent inflammatory episodes despite receiving the highest tolerable doses (1.5 to 2.0 mg/day) of colchicine, which are considered to be within the effective range.

The majority of FMF patients have autosomal recessive inheritance associated with mutations in the *MEFV* gene, which encodes pyrin protein [1]. FMF-related *MEFV* mutations, which affect pyrin-mediated regulation of caspase 1 activity in the inflammasomes, are associated with increased IL-1β production in mice and humans [1]. Therefore, inhibition of IL-1 activity may decrease both frequency and severity of acute attacks in patients with FMF. Several reports of patients with FMF being successfully treated with agents blocking IL-1 activity, mainly with daily injections of the recombinant form of IL-1 receptor antagonist (IL-1Ra), anakinra, have confirmed the critical role of IL-1 in the pathogenesis FMF [5, 6].

The objective of this study was to evaluate the efficacy and safety of canakinumab, a fully human anti-IL-1β monoclonal antibody with a half-life of approximately 4 weeks, that binds to human IL-1β and neutralizes its proinflammatory effects, in adolescent and adult patients with FMF, who are resistant or intolerant to higher doses of colchicine.

## Methods

The present study was an investigator-initiated, open-label exploratory trial that included adolescent and adult FMF patients with active disease despite receiving the highest tolerable doses of colchicine (1.5 to 2.0 mg/day). All patients had a typical type I phenotype, fulfilling the criteria for FMF diagnosis [7], along with at least one of the exon 10 mutations in the *MEFV* gene. Patients with end-organ dysfunction due to secondary amyloidosis, active tuberculosis or any other infectious diseases, or a history of malignancy within the last 5 years were excluded from the study.

Colchicine-compliant patients with a history of one or more attacks per month within 3 months before the screening were eligible to enter the first 30-day run-in period. Patients who had at least one attack during that period advanced to a second 30-day period, and they received their first dose of canakinumab upon the first attack they experienced during this second 30-day run-in period.

The treatment period started with the first injection, and patients received a total of three subcutaneous injections of canakinumab 150 mg at 4-week intervals. The canakinumab dose could be increased to 300 mg, if an attack occurred between the first and second doses. Stable doses of colchicine (1.5 to 2.0 mg/day) were allowed throughout the study without any dose modification, and compliance was followed tightly throughout the study. After the 12-week treatment period, the patients were subsequently followed for up to 2 months or until the next attack.

FMF attacks were confirmed by presence of fever, clinical findings of serositis/arthritis, and elevated C-reactive protein (CRP) levels. Details of each attack (duration, type, severity, maximum body temperature) were recorded in diaries. Diaries were dispensed to the colchicine-resistant FMF (crFMF) patients at each visit to record the occurrence of any attacks between the scheduled visits.

The primary outcome measure was the proportion of patients with 50 % or more reduction in time-adjusted frequency of attacks. Due to the unequal pre-treatment and treatment periods, attack rates were adjusted to the 84-day treatment period compared with the pre-treatment periods. Secondary outcome measures included the percentage of patients with no attacks in the treatment period, time to next attack after the last canakinumab administration, changes in quality of life assessed by the 36-item short-form health survey (SF-36), and serum levels of CRP and serum amyloid A (SAA) proteins. Physicians’ and patients’ global assessments of control of FMF since the last visit and response to treatment at the end of the treatment period were also measured using a modified 5-point scale [8]. All adverse events and laboratory values were recorded at each visit for the assessment of safety of canakinumab treatment.

Istanbul Faculty of Medicine Ethics Committee and Ministry of Health approved the study protocol, and all patients provided written informed consent before the screening. Exploratory analyses were performed using descriptive statistics.

## Results and discussion

A total of 13 patients with crFMF were screened and included in the first 30-day run-in period. Nine patients (median age 22 years, range, 12 to 34) had ≥1 attack during that period, advancing to the second 30-day period, and began their treatment period by starting canakinumab treatment at the first observed attack within this period. Patient baseline demographic characteristics and *MEFV* genotypes are summarized in Additional file 1: Table S1.

All nine patients in the treatment period achieved the primary endpoint of ≥50 % reduction in frequency of attacks compared with the time-adjusted pre-treatment frequency of attacks. During the treatment period, only one patient, who was p.Met694Val homozygous and receiving 2 mg/day colchicine, had an attack of peritonitis on day 54. No patient qualified for a canakinumab dose increase between the first and second injections. The time-adjusted frequency of attacks over 84 days observed in the screening and run-in periods, including the baseline attack (median 3.29, range 2.47 to 4.2), decreased dramatically during the treatment period (median 0, mean 0.11).

**Fig. 1:**
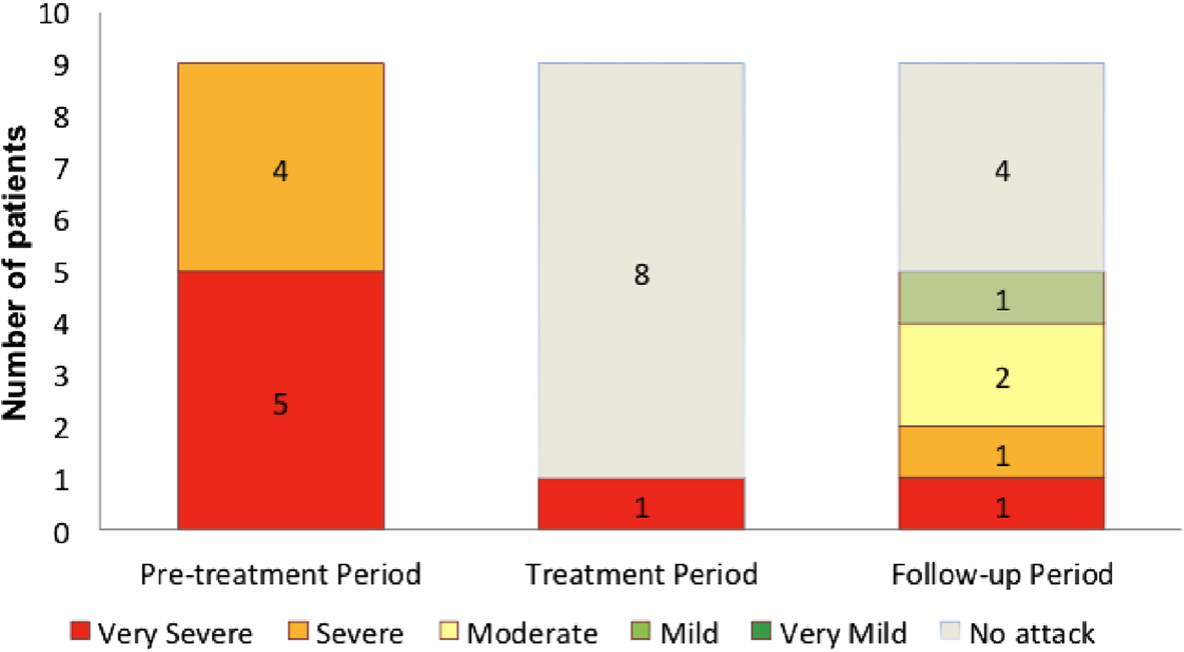
Number and severity of familial Mediterranean fever attacks observed throughout the study period. Severity of the attack was assessed according to patients’ assessment in relation to their previous experiences

Five patients, all p.Met694Val homozygous and receiving 2 mg/day colchicine, subsequently experienced an attack within the 2-month follow up, which occurred at a median 71 days (range 31 to 78 days) after the last canakinumab injection (Fig. 1). Median baseline CRP and SAA levels (58 mg/L and 162 mg/L, respectively) on day 1 of canakinumab administration normalized (2.5 mg/L and 5.8 mg/L, respectively) by day 8, and remained low throughout the study (Additional file 2: Figure S1). No other significant laboratory abnormalities were noted.

**Fig. 2:**
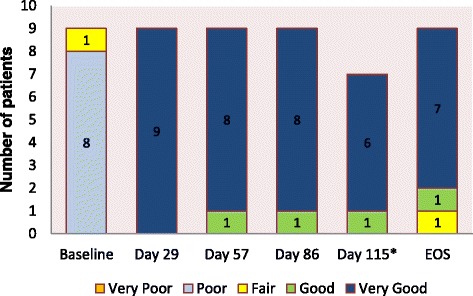
Physician’s global assessment of control of familial Mediterranean fever over the previous month. *EOS* end of study

The median SF-36 physical and mental component scores increased dramatically at day 8 compared with baseline scores and continued to improve throughout the treatment period (Additional file 3: Figure S2). Compared with baseline, the physician’s and patient’s global assessment of crFMF control improved with canakinumab treatment, and overall treatment response was reported as being very good both by physicians (for all patients) and patients (seven of nine patients) at the end of study (Fig. 2).

Eight patients reported at least one adverse event; headache (n = 4) and upper respiratory tract infection (n = 2) were the only adverse events reported by more than one patient (Table 1). All adverse events were mild or moderate except one, which was a severe headache. None of the patients discontinued the trial due to an adverse event. A mild, local injection-site reaction was recorded in two patients on at least one occasion.

**Table 1:** Adverse events observed in nine patients during the canakinumab treatment period

	Number (%)
Adverse events	8 (88.9)
Ankle fracture	1 (11.1)
Anxiety	1 (11.1)
Headache	4 (44.4)
Hidradenitis^a^	1 (11.1)
Pregnancy	1 (11.1)
Pruritus	1 (11.1)
Tooth infection	1 (11.1)
Upper respiratory tract infection	2 (22.2)
Vomiting	1 (11.1)

^a^This patient had a medical history of hidradenitis, and she had no serious exacerbation during canakinumab treatment

One of the patients became pregnant during the treatment period after the third canakinumab dose and gave birth to a healthy son.

This open-label pilot trial showed that monthly canakinumab 150 mg subcutaneous injections prevented attacks in patients with crFMF, and only one of nine patients experienced an attack during canakinumab treatment. The safety profile of canakinumab in this small group of patients was similar to that of larger controlled trials in other hereditary autoinflammatory conditions [8–10].

Colchicine has long been used to prevent inflammatory attacks and reduce the risk of secondary amyloidosis in patients with FMF. Despite the efficacy of colchicine, some manifestations, such as arthritis, are less responsive to colchicine [11]. Furthermore, 5−10 % of patients are non-responders to colchicine [12], and the number of FMF patients who are either intolerant or resistant to colchicine and continue to experience frequent and/or severe inflammatory attacks is increasing [13].

Given the association of type and number of *MEFV* variations with increased production of IL-1β, several case reports of patients treated with either anakinra or canakinumab have suggested the potential efficacy of IL-1 blockade in colchicine-resistant patients and those with amyloidosis [5, 6]. Additionally, in a recent randomized, double-blind, alternating-treatment trial the frequency of attacks decreased with weekly injections of rilonacept, another anti-IL-1 drug, consisting of humanized IL-1 type 1 receptor, IL-1 receptor accessory protein and the Fc portion of IgG1 [14]. All three drugs block IL-1 beta activity, but there are some differences, mainly resulting from the half-lives of these drugs, the shortest being for anakinra, requiring injections at least once daily, and the longest being for canakinumab, requiring monthly or bi-monthly injection intervals, which may affect the quality of life of patients. There is no study comparing the dynamics of IL-1 beta secretion in patients with crFMF with those in patients with cryopyrin-associated periodic fever syndrome (CAPS) [8, 9]. Therefore, in this pilot trial, we aimed to test the efficacy and safety of canakinumab in patients with crFMF by 4-weekly administration intervals for 3 months and followed them up for 2 months to observe any recurrence of inflammatory attacks. In this follow-up period there was an inter-individual variability in the timing of recurrent attacks, with a range of 31 to 78 days after the last dose in five patients, and possibly reflecting differences in their inflammatory activity. Randomized controlled trials are expected to provide further data about the optimum dosage and administration intervals of canakinumab in patients with crFMF.

The limitations of this small exploratory study were its open-label design, relatively short treatment period, and lack of formal definitions for disease severity and colchicine resistance. However, inclusion of mainly patients with FMF with two penetrant mutations and ≥1 attack per month despite receiving the highest tolerable doses of colchicine, together with confirmation of attacks and colchicine-compliance during the run-in period, represent a real-world patient cohort with few treatment options.

## Conclusions

In conclusion, the results of the present pilot trial suggest that canakinumab may be an effective and safe treatment option for colchicine-resistant and colchicine-intolerant patients with FMF, and warrant further investigations to explore its efficacy, safety and optimum dosage and administration intervals in this subset of FMF patients.

## Additional files

Additional file 1: Table S1. Demographics and baseline characteristics of the patients with familial Mediterranean fever (FMF) during the enrollment into the treatment period (n = 9). *CRP* C-reactive protein, *ESR* erythrocyte sedimentation rate, *SAA* serum amyloid A. (DOCX 16 kb) Additional file 2: Figure S1. Serum C-reactive protein (*CRP*) and serum amyloid A (*SAA*) measurements during the treatment and follow-up periods. *EOS* end of study. (DOCX 61 kb) Additional file 3: Figure S2. Short form-36 (*SF-36*) physical component summary (*PCS*) and mental component summary (*MCS*) scores during the treatment and follow-up periods. *EOS* end of study. (DOCX 53 kb)

## Abbreviations

crFMF: colchicine-resistant familial Mediterranean fever
CRP: C-reactive protein
FMF: familial Mediterranean fever
IL-1: interleukin-1
SAA: serum amyloid A
SF-36: Short form-36
