# Supplementary material for: Efficacy and safety of canakinumab in adolescents and adults with colchicine-resistant familial Mediterranean fever
Source: Arthritis Res Ther. 2015 Sep 4;17(1):243. doi: 10.1186/s13075-015-0765-4 (PMC4559892; doi:10.1186/s13075-015-0765-4)
Supplement: Additional file 1: Table S1. — Demographics and baseline characteristics of the patients with familial Mediterranean fever (FMF) during the enrollment into the treatment period (n = 9). CRP C-reactive protein, ESR erythrocyte sedimentation rate, SAA serum amyloid A. (DOCX 16 kb) [file 13075_2015_765_MOESM1_ESM.docx]

**Additional file 1**

**Table S1.** Demographics and baseline characteristics of the FMF patients during the enrollment into the treatment period (n=9).

Sex 7 female, 2 male

Mean age, years (±SD) 22.33 ± 6.34

Mean weight, kg (± SD) 63.22 ± 16.29

Mean height, cm (± SD) 163.25 ± 9.92

Median CRP, mg/L 58

Median ESR, mm/hour 34

Median SAA, mg/L 162

Time-adjusted attack frequency* 3.29 (2.47-4.2)

MEFV mutations p.Met694Val homozygous (6)

p.Met680Ile/p.Met694Val compound heterozygous (1)

p.Met694Val heterozygous (1)

p.Met680Ile heterozygous (1)

Highest tolerable colchicine dose 1.5 (2 patients) to 2 mg/day (7 patients)

*Time adjusted attack frequency/84 days observed in screening and run-in up to and including baseline attack.
