# Supplementary material for: Efficacy and safety of canakinumab in adolescents and adults with colchicine-resistant familial Mediterranean fever
Source: Arthritis Res Ther. 2015 Sep 4;17(1):243. doi: 10.1186/s13075-015-0765-4 (PMC4559892; doi:10.1186/s13075-015-0765-4)

**Additional file 2**

**Figure S1.** Mean serum CRP and SAA measurements during the treatment and follow-up periods.


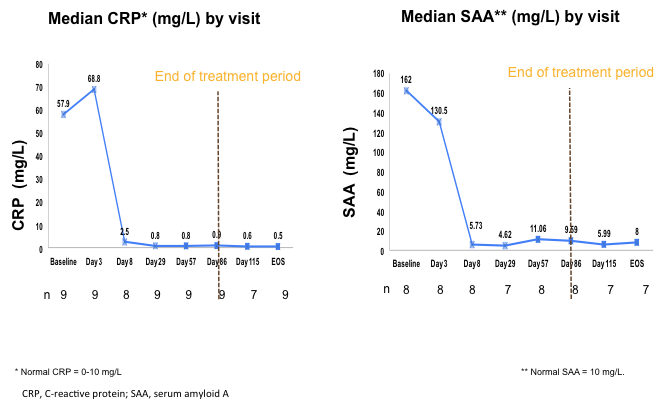

Supplement: Additional file 2: Figure S1. — Serum C-reactive protein (CRP) and serum amyloid A (SAA) measurements during the treatment and follow-up periods. EOS end of study. (DOCX 61 kb) [file 13075_2015_765_MOESM2_ESM.docx]
