# Supplementary material for: Efficacy and safety of canakinumab in adolescents and adults with colchicine-resistant familial Mediterranean fever
Source: Arthritis Res Ther. 2015 Sep 4;17(1):243. doi: 10.1186/s13075-015-0765-4 (PMC4559892; doi:10.1186/s13075-015-0765-4)

Additional file 3

**Figure S2.** SF-36 Physical Component Summary (PCS) and Mental Component Summary (MCS) Scores during the treatment and follow-up periods.


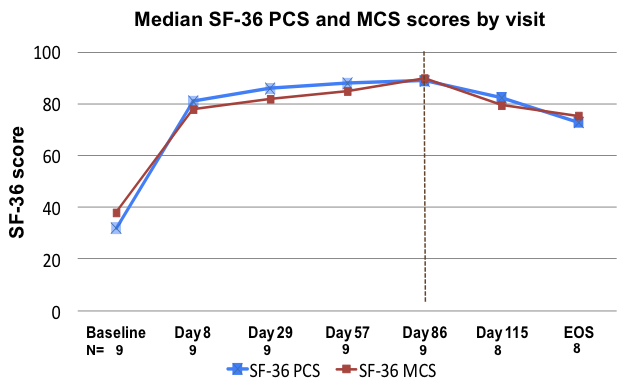

Supplement: Additional file 3: Figure S2. — Short form-36 (SF-36) physical component summary (PCS) and mental component summary (MCS) scores during the treatment and follow-up periods. EOS end of study. (DOCX 53 kb) [file 13075_2015_765_MOESM3_ESM.docx]
